# Methods for dealing with discrepant records in linked population health datasets: a cross-sectional study

**DOI:** 10.1186/1472-6963-7-12

**Published:** 2007-01-30

**Authors:** Christine L Roberts, Charles S Algert, Jane B Ford

**Affiliations:** 1 The Kolling Institute of Medical Research, St Leonards 2065, Australia; 2 The George Institute for International Health, Camperdown 2050, Australia

## Abstract

**Background:**

Linked population health data are increasingly used in epidemiological studies. If data items are reported on more than one dataset, data linkage can reduce the under-ascertainment associated with many population health datasets. However, this raises the possibility of discrepant case reports from different datasets.

**Methods:**

We examined the effect of four methods of classifying discrepant reports from different population health datasets on the estimated prevalence of hypertensive disorders of pregnancy and on the adjusted odds ratios (aOR) for known risk factors. Data were obtained from linked, validated, birth and hospital data for women who gave birth in a New South Wales hospital (Australia) 2000–2002.

**Results:**

Among 250173 women with linked data, 238412 (95.3%) women had perfect agreement on the occurrence of hypertension, 1577 (0.6%) had imperfect agreement; 9369 (3.7%) had hypertension reported in only one dataset (under-reporting) and 815 (0.3%) had conflicting types of hypertension. Using only perfect agreement between birth and discharge data resulted in the lowest prevalence rates (0.3% chronic, 5.1% pregnancy hypertension), while including all reports resulted in the highest prevalence rates (1.1 % chronic, 8.7% pregnancy hypertension). The higher prevalence rates were generally consistent with international reports. In contrast, perfect agreement gave the highest aOR (95% confidence interval) for known risk factors: risk of chronic hypertension for maternal age ≥40 years was 4.0 (2.9, 5.3) and the risk of pregnancy hypertension for multiple birth was 2.8 (2.5, 3.2).

**Conclusion:**

The method chosen for classifying discrepant case reports should vary depending on the study question; all reports should be used as part of calculating the range of prevalence estimates, but perfect matches may be best suited to risk factor analyses. These findings are likely to be applicable to the linkage of any specialised health services datasets to population data that include information on diagnoses or procedures.

## Background

Population health datasets (PHDS) provide a valuable tool for epidemiological and health services research with the capacity to address health care, policy and planning issues[1,2]. PHDS include population-based collections of data relevant to health outcomes and health services that are available in unit-record form. Advantages of using PHDS for research include the ability to describe the total health burden of disease in the population and to assess risk factors and causal pathways of outcomes[3]. If the population is large enough, study hypotheses that involve infrequent outcomes or exposures can be addressed as well as hypotheses that involve small effects of common exposures and outcomes[4]. Furthermore, research using PHDS is less resource-intensive than follow-up of large samples over time and minimises loss to follow-up and recall bias particularly regarding sensitive issues[3,5]. While the population coverage and availability of PHDS make them an attractive and inexpensive resource for research, there are limitations relating to the completeness and validity of data in studies utilising single datasets[3]. Linkage of PHDS can help overcome some of the disadvantages of a single cross-sectional dataset such as under-ascertainment or misclassification of exposures and outcomes, and enables the capture of important longitudinal outcomes including mortality and major morbidities [6-9]. On the other hand, record linkage allows the possibility of discrepant case reports if exposure or outcome information are collected on more than one PHDS. We experienced this situation in a study utilising linked PHDS to examine hypertensive disorders of pregnancy where maternal hypertension status was recorded in both a perinatal data collection and in hospital discharge data[10].

'Hypertensive disorders of pregnancy' encompass two different, but related, conditions[11,12]. Chronic hypertension predates the pregnancy or has onset prior to 20 weeks gestation. The main risk factor for chronic hypertension is advancing maternal age [10-12]. Pregnancy hypertension, arising de novo from 20 weeks of gestation, is a syndrome ranging from hypertension alone (gestational hypertension) through proteinuria and multi-organ dysfunction (preeclampsia) to seizures (eclampsia) [10-12] Some women with chronic hypertension develop superimposed preeclampsia. Multiple (multifetal) pregnancy is a major risk factor for pregnancy hypertension [10-12].

Previous PHDS studies examining outcomes reported on more than one dataset have accepted any report of the condition of interest as a "case" [13-15]. However, for hypertensive disorders of pregnancy, classifying hypertension as a yes/no variable is not clinically useful because the different types of hypertension in pregnancy have different risk factors, care requirements and adverse event probabilities [10-12]. Other methods of dealing with discrepant case reports from more than one dataset could not be identified in the published literature, although capture-recapture methods have been used to estimate completeness when data sources are independent [16-18]. Discrepant case reports raise the possibility of misclassifying types of hypertension as well as misclassification of hypertensive status generally. Therefore, the aim of this study was to examine alternative methods of classifying discrepant case reports from different population health datasets, and to assess the impact on estimates of disease prevalence and on the strength of association with known risk factors.

## Methods

The study population included all women, discharged from hospital following birth in New South Wales (NSW) Australia, 1 January 2000 to 31 December 2002. Only 1% of women have homebirths[19]. Data for the study were obtained from existing NSW Department of Health computerized datasets: the Midwives Data Collection (MDC) and the Inpatient Statistics Collection (ISC). The MDC is a legislated population-based surveillance system covering all NSW births ≥20 weeks gestation or ≥400 g birthweight, that includes information on maternal characteristics, pregnancy, labour, delivery and infant outcomes[19]. The MDC relies on the attending midwife or doctor to complete a notification form when the birth occurs [19]. A copy of the MDC notification form is retained in the medical records. The ISC is a census of all NSW inpatient hospital separations (public and private); data are coded from the medical records according to the 10^th ^revision of the International Classification of Diseases (ICD10)[20,21]. Hospital coders use all available information in the medical record, including the MDC form, to code diagnoses and procedures. Only ISC records for the birth admission were used in this study. Both data sets have been validated against the medical records in separate studies at different times [22,23]. In these validation studies, the medical records of randomly selected birth admissions were reviewed and information was abstracted by a health information manager and a clinical nurse consultant in midwifery [22,23]. The validation data were then compared with the data on the MDC or ISC, using the validation data as the gold standard. The NSW Department of Health performed record linkage of the two datasets and produced de-identified linked birth and hospital records. Linkage proportions for the two datasets were over 97 percent[24]. This study was approved by an institutional ethics committee.

Pregnancy hypertension and chronic hypertension are reported on both the MDC and the ISC. The conditions are not mutually exclusive, as some women have chronic hypertension with superimposed pre-eclampsia. The MDC uses a check-box format for hypertension reporting. In the ISC, an ICD10 code for a hypertensive disorder of pregnancy (O10–O16)[21] in any of the 21 available fields was accepted as a diagnosis. Gestational hypertension, pre-eclampsia and eclampsia were grouped as pregnancy hypertension.

The true positive rate for case identification of the type of hypertension is dependent on both the sensitivity and the specificity of the reporting on the datasets and the prevalence of the condition. The validation studies of the MDC and ISC found both datasets suffer from under-reporting of hypertension (sensitivities 50–86%) but that specificities were very high (99.1–99.8%)[22,23]. Because hypertensive disorders of pregnancy are not common conditions, the number of false positives could still be non-negligible relative to the number of true positives.

Alternative methods of classifying inconsistent and discrepant reports from the two datasets were examined. The occurrence and type of hypertension reporting on the two datasets were classified in the following ways (Table 1):

**Table 1:** Consistency of hypertension reporting between the Midwives Data collection (MDC) and Inpatients Statistics Collection (ISC), New South Wales, 2000–2002.

**Hypertension type by ISC**	**Hypertension type by MDC**			
	**Chronic**	**Pregnancy**	**Chronic + preclampsia**	**No hypertension**
**Chronic**	531^1^	150^4^	80^2^	233^3^
**Pregnancy**	665^4^	12144^1^	350^2^	5192^3^
**Chronic + preeclampsia**	109^2^	178^2^	81^1^	36^3^
**Unspecified**	125^2^	717^2^	18^2^	1132^3^
**No hypertension**	522^3^	2177^3^	77^3^	225656^1^

Agreement in reporting between the two datasets

1. perfect agreement

2. imperfect agreement

3. under-reporting

4. conflicting reports on the type of hypertension

1. Totally consistent reports on hypertension occurrence and type (perfect agreement)

2. Partially consistent reports, such that if one dataset recorded one type of hypertension and the other recorded both only the common type was accepted, and where the ISC reported unspecified hypertension and the MDC specified a type then the MDC report was accepted (imperfect agreement).

3. One dataset reported hypertension and the other did not report any hypertension, then the hypertension report was accepted (under-reporting), or

4. Conflicting reports on the type of hypertension, where chronic hypertension was reported on one dataset and pregnancy hypertension on the other (conflicting). The hypertension of interest in any analysis was accepted to be true. So in the analysis of chronic hypertension, the conflicting cases were accepted as chronic hypertension and similarly for the pregnancy hypertension analysis.

These categories were then sequentially combined to make four alternative methods for classifying a woman as a "case" (having the hypertension type of interest): 1) only perfect agreement counted 2) perfect agreement + imperfect agreement counted 3) perfect agreement + imperfect agreement + under-reporting agreement counted 4) any report (all four categories used to count hypertensive cases). These four alternative methods were assessed to determine the effect on both the prevalence of hypertension and on the strength of association of known risk factors for hypertension.

The resulting prevalence rates of chronic hypertension and pregnancy hypertension, using the above four classification methods as well as the rates from the individual datasets, were compared. We then modeled hypertensive status as a dependent variable, with adjustment for risk factors, focusing on a type-specific risk factor: maternal age for chronic hypertension and multiple pregnancy for pregnancy hypertension. The risk factor information was obtained from the MDC and is accurately reported: maternal age has 97% agreement with the medical record (kappa not calculated) and multiple pregnancy has 99.5% agreement (kappa 0.89). Because misclassification of an outcome or exposure usually (although not invariably) biases measures of association towards the null, we hypothesized that the magnitude of risk (measured as adjusted odds ratios [aOR] with 95 percent confidence intervals [CI]) for known risk factors would move further from unity with less misclassification. The goodness-of-fit of the logistic regression models was assessed with the Hosmer Lemeshow Test (all p-values >0.5).

Data from previous validation studies were used to calculate "corrected" prevalence rates for the MDC and ISC, for comparison purposes. The corrected prevalence was calculated by multiplying the crude rate from the MDC or ISC by the positive predictive values (PPV) from the validation studies and dividing by the sensitivity[22,23].

## Results

Among the 250173 women with linked data available, 238412 (95.3 percent) women had perfect agreement on the occurrence and type of hypertension, 1577 (0.6 percent) had imperfect agreement; 9369 (3.7 percent) were classified as under-reported and 815 (0.3 percent) had conflicting reports on the type of hypertension (Table 1).

The effect on the estimated prevalence rates of using different methods for classifying hypertension status was broadly similar for both chronic hypertension and pregnancy hypertension (Table 2). For both of these conditions, restricting "cases" to those where there was total agreement between the MDC and the ISC resulted in the smallest prevalence rate, and using the method which included all reports including inconsistent and conflicting reports resulted in the highest prevalence rates. Using the MDC or ISC alone, or the other classification methods, resulted in intermediate prevalence rates. For chronic hypertension, however, the prevalence estimate based on the MDC was nearly as high as that based on any report including conflicting reports. Chronic hypertension was rare, with a maximum prevalence estimate of only 1.05 percent even if discrepant reports were accepted. Pregnancy hypertension was uncommon, with a maximum prevalence of 8.71 percent if conflicting reports were accepted.

**Table 2:** Effect of different methods of classifying discrepant reports of hypertension on the prevalence and risk factors for hypertensive disorders of pregnancy

			*Type of agreement included in analysis*			
	MDC Dataset only	ISC Dataset only	*Perfect Agreement*	*Perfect + Imperfect Agreement*	*Perfect + Imperfect +* * Under-reporting*	*Perfect + Imperfect + * *Under-reporting + Conflicting*
	N = 250173	N = 250173	*N = 238412*	*N = 239989*	*N = 249358*	N = 250173
**Chronic hypertension**						
	n (%)	n (%)	n (%)	n (%)	n (%)	n (%)
Prevalence	2558 (1.02)	1398 (0.56)	612 (0.26)	944 (0.39)	1812 (0.73)	2627 (1.05)
						
Age*	aOR (95%CI)	aOR (95%CI)	aOR (95%CI)	aOR (95%CI)	aOR (95%CI)	aOR (95%CI)
<20 years	0.52 (0.39, 0.70)	0.41 (0.27, 0.64)	0.25 (0.11, 0.57)	0.38 (0.22, 0.66)	0.51 (0.36, 0.73)	0.52 (0.39, 0.69)
20–24 years	0.64 (0.55, 0.75)	0.59 (0.47, 0.73)	0.38 (0.26, 0.56)	0.50 (0.38, 0.66)	0.64 (0.53, 0.77)	0.65 (0.56, 0.76)
25–29 years	1.00 (Referent)	1.00 (Referent)	1.00 (Referent)	1.00 (Referent)	1.00 (Referent)	1.00 (Referent)
30–34 years	1.11 (1.00, 1.23)	1.16 (1.00,1.33)	1.13 (0.91, 1.40)	1.08 (0.91, 1.29)	1.13 (1.00, 1.28)	1.12 (1.01, 1.24)
35–39 years	1.57 (1.40, 1.76)	1.88 (1.61, 2.18)	2.05 (1.64, 2.56)	1.91 (1.59, 2.29)	1.70 (1.49, 1.95)	1.62 (1.45, 1.81)
≥40 years	2.69 (2.29, 3.15)	3.35 (2.73, 4.12)	3.95 (2.94, 5.30)	3.58 (2.80, 4.56)	2.78 (2.30, 3.35)	2.59 (2.20, 3.04)
**Pregnancy hypertension**						
	n (%)	n (%)	n (%)	n (%)	n (%)	n (%)
Prevalence	15972 (6.38)	18771 (7.50)	12225 (5.13)	13488 (5.62)	20970 (8.41)	21785 (8.71)
						
Plurality†	aOR (95%CI)	aOR (95%CI)	aOR (95%CI)	aOR (95%CI)	aOR (95%CI)	aOR (95%CI)
Multiple	2.35 (2.09, 2.64)	2.81 (2.53, 3.13)	2.78 (2.45, 3.15)	2.60 (2.30, 2.95)	2.74 (2.46, 3.04)	2.71 (2.44, 3.01)
Singleton	1.00 (Referent)	1.00 (Referent)	1.00 (Referent)	1.00 (Referent)	1.00 (Referent)	1.00 (Referent)

aOR = adjusted odds ratio, MDC = Midwives data Collection, ISC = Inpatients Statistics Collection

* Age-associated risk for chronic hypertension adjusted for parity, diabetes and smoking

† Plurality-associated risk for pregnancy hypertension adjusted for age, parity, diabetes, smoking and chronic hypertension

Table 3 shows the crude prevalence rates for chronic hypertension and pregnancy hypertension, as reported on the MDC and ISC, and the corrected rates after allowing for the estimated PPV and sensitivity from the previous validation studies. The crude rates have narrow 95% confidence intervals for the sampling error, and three of the four corrected rates lie outside of the confidence intervals. The MDC may over-estimate the prevalence of chronic hypertension, since correction reduced the estimated prevalence from 1.02 percent to 0.91 percent, a relative decrease of 11%. The chronic hypertension estimate from the ISC is unchanged after correction. For pregnancy hypertension, the corrected estimates of prevalence from the MDC and ISC converge. Both datasets show a corrected prevalence of around 9 percent, similar to the 8.71 percent prevalence determined if any report, including conflicting reports, of pregnancy hypertension is counted.

**Table 3:** Crude and "corrected" rates of hypertensive disorders as reported in the Midwives Data collection (MDC) and Inpatients Statistics Collection (ISC)

**Dataset**	**Reported Prevalence (%)**	**95% CI**	**Estimated PPV %**	**Estimated sensitivity %**	**"Corrected" prevalence**‡
	**Chronic hypertension**				
MDC	1.02	0.98, 1.06	55.6*	62.5*	0.91
ISC	0.56	0.53, 0.59	85.7†	85.7†	0.56
	**Pregnancy hypertension**				
MDC	6.38	6.29, 6.48	90.1*	66.7*	8.61
ISC	7.50	7.40, 7.61	81.0†	58.6†	9.11

* Positive predictive value (PPV) and sensitivity estimates from a previous validation study of 1680 women [22]

† PPV and sensitivity estimates from a previous validation study of 490 women [23]

‡ "corrected" prevalence = reported prevalence × PPV/sensitivity

95% CI = 95 percent confidence interval

For the rare condition of chronic hypertension, the aOR's for maternal age categories were sensitive to the method chosen for classifying cases (Table 2). For the youngest and oldest women, the aOR for chronic hypertension was farthest from unity when chronic hypertension was classified using a perfect match between the MDC and ISC datasets (<20 years: aOR = 0.25; > = 40 years: aOR = 3.95). Using the combination of perfect and imperfect matches also moved the aOR for chronic hypertension for the youngest and oldest women further from unity than using either the MDC or ISC alone, but by a smaller amount. The other classification methods, which included under-reporting and conflicting reports, resulted in aOR's which were similar to aOR's using the MDC reports of chronic hypertension, and were closer to unity than aOR's based on ISC reports. Using the ISC reports alone for this condition resulted in aOR's that were noticeably further from unity than if the MDC alone was used.

The aOR's for chronic hypertension should not have been noticeably shifted further from unity by misclassification of pregnancy hypertension, as in the ISC maternal age was only weakly associated with pregnancy hypertension (<20 years: aOR = 0.96 (95 percent CI: 0.89, 1.04); ≥40 years: aOR = 1.22 (95 percent CI: 1.07, 1.42).

Pregnancy hypertension showed less sensitivity to how the condition was classified when examining the risk associated with multiple pregnancy (Table 2). Using the ISC reports alone resulted in an aOR (2.81) that was further from unity than using the MDC reports alone (2.35), and was in fact the maximum aOR of pregnancy hypertension for multiple birth. The aOR based upon perfect matches (2.78) was similar to that using the ISC alone. Adding imperfect matches, under-reporting and conflicting matches resulted in movements of the aOR towards unity.

The aOR's for pregnancy hypertension should not have been shifted further from unity by misclassification of chronic hypertension, as the prevalence of this condition is low and in the ISC, multiple pregnancy was not significantly associated with chronic hypertension (aOR = 1.25 (95 percent CI: 0.88, 1.77).

## Discussion

This study demonstrates the importance of choosing a method of classifying outcomes of interest that is appropriate to the purpose of the analysis. For many population cross-sectional studies, low sensitivities and under-enumeration of cases may be a real concern. Using data from more than one dataset offers the opportunity to identify more cases, albeit with uncertain effects on specificity and PPV. Examination of possible causal factors may be a secondary consideration and potential under-estimation of risks due to misclassification of the outcome not a prime concern. But if examination of potential risk factors is the main reason for a study, care needs to be taken to minimize the possibilities for misclassification of outcomes and the resultant under-estimation of risks. This is particularly true when the outcome of interest is rare, such that the number of true positives and false positives may be similar. When the incidence or prevalence of a condition is less than one percent, false positives may outnumber true positives even if specificity is greater than 99 percent. In this study, the odds of having chronic hypertension for a woman aged 40 years or more would have been underestimated by 40 percent if perfect and imperfect matches plus under-reporting were used to classify cases as opposed to using only perfect matches (aOR = 2.78 vs 3.95).

Using two separate datasets to try to capture cases is known to increase sensitivity[6,7,9] and it is likely in this instance that relying on the ISC alone for chronic hypertension or the MDC alone for pregnancy hypertension would have resulted in estimated rates of these conditions that were too low. Reliance on the MDC alone might result in an over-estimate of the prevalence of chronic hypertension. Use of the different classification methods does not necessarily provide a more accurate estimate of prevalence than use of a single dataset, but it does provide a range of estimates that reflects possible misclassification error, providing information that is not available from the usual 95% confidence interval which only takes into account sampling error. For large datasets such as the ones used in this study, sampling error may be small relative to misclassification error, and narrow 95% confidence intervals may give a false sense of certainty about estimated prevalence rates. Calculation of corrected rates, using data on PPV and sensitivity of reporting from validation studies, can provide additional estimates which can help to inform a choice as to the best estimate of the true rates. The corrected rates for this study are generally consistent with published population rates of chronic and pregnancy hypertension[15,25-28]. The reliability of the correction to the crude rates depends on the reliability of the estimates of the PPV and sensitivity for reporting of the conditions. These estimates are less reliable for the rarer condition of chronic hypertension, where the ISC estimates of PPV and sensitivity were based on a sample population of 490 women in the ISC validation study[23].

Capture-recapture methods have been used elsewhere to evaluate the completeness of case ascertainment and estimate prevalences corrected for under-ascertainment [16-18,29]. However, the ISC and MDC violate the crucial assumption of independence of the data sources as the MDC notification form is available for abstracting data for the ISC. Thus a case identified in the MDC may be more likely to be identified in the ISC than those not identified by the MDC. This positive dependence would result in the number of cases in the population being under-estimated if a capture-recapture method was used [16,17].

Without estimates of the PPV and sensitivity of each method of classifying outcomes, it is not certain which of the classification method results in the most accurate estimates for prevalence. However, for a variable which is already known to be a risk factor for the outcome of interest, movements in the aOR away from unity should reflect a higher rate of true positives and increased PPV. Comparing the aOR's for different methods of classifying reports makes it possible to assess which method, and which dataset, is likely to have relatively better PPV's. For both of the conditions in this study, the ISC appeared to more accurately identify cases than the MDC. This is consistent with other findings that hospital discharge data are more accurately reported than birth data, which may be because hospital reporting is tied to compensation[6,7,9].

An important aspect of the design of this study was to select risk factors which were specific to either pregnancy hypertension or chronic hypertension in pregnant women. The aOR's for these risk factors would only move further away from unity if more of the relevant type of hypertension was identified.

It is possible that cases of hypertension reported on both databases had a higher proportion at the more severe end of the hypertension spectrum. However, we could not identify any studies that support this conjecture. In 1992, Iezzoni suggested that among elderly hospitalised patients, those who were severely ill and in the process of dying have more severe acute conditions and complications that take precedence over the coding of chronic diseases [30]. However this does not answer the question of whether more severe conditions (such as severe preeclampsia) are likely to appear in any or multiple databases. Concurrent validation of data sources is required to confirm whether more severe cases have a higher probability of capture on multiple data sources. However, even if this did affect outcome classification, it is not clear that it would affect the aOR for risk factors of the outcome. In this study, use of the ISC alone captured many more cases of pregnancy hypertension than the subgroup of cases captured only if they were reported on both the ISC and the MDC. But the aOR's of pregnancy hypertension for multiple birth were nearly identical for the two classification methods.

Although this study is based on perinatal data, the findings are likely to be applicable to the linkage of any specialised health services datasets that include information on diagnoses or procedures. Increasing linkage of such datasets and additional linkages with population health registries, such as cancer, stroke, coronary heart disease and pharmaceuticals [31-34], will increase the need for assessments of the usefulness and accuracy of the linked data. Ideally validation of multiple datasets should occur at the same time so that the impact of accepting reports from more than one source can be evaluated.

## Conclusion

In conclusion, we have demonstrated that how cases are classified, when more than one dataset is used to identify cases, can impact on estimates of risk for study factors. A more restrictive method for classifying cases which minimizes misclassification may be warranted when risk assessment is a primary concern for a study. One approach to comparing the relative strength of the PPVs of different datasets and of different methods of classifying reported cases of the condition of interest may be to examine how the adjusted OR (for a previously established risk factor) is affected when different methods are used.

## List of Abbreviations

PHDS Population health datasets

NSW New South Wales

MDC Midwives Data Collection

ISC Inpatient Statistics Collection

ICS10 International Classification of Diseases, 10^th ^revision

PPV Positive Predictive Value

aOR adjusted Odds Ratio

95 percent CI 95 percent confidence interval

## Competing interests

The author(s) declare that they have no competing interests.

## Authors' contributions

CLR conceived the study, undertook the data analysis and drafted the manuscript. CSA participated in the design of the study, advised on the statistical analysis and helped to draft the manuscript. JBF participated in the study design and interpretation of the data. All authors read and approved the final manuscript.

## Pre-publication history

The pre-publication history for this paper can be accessed here:


